# Palo Santo (*Bursera graveolens*) essential oil nanoemulsion: toxicological, antinociceptive and antimicrobial potential against bovine mastitis–associated strains

**DOI:** 10.1007/s10482-026-02322-w

**Published:** 2026-04-30

**Authors:** Amanda Marques Figueiredo, Sabrina Valadão Vilela dos Reis, Alex Alves Rodrigues, Jhuan Luiz Silva, Isabelly Moreira Colombo, Leandra Maria Diniz Batista Leite, João Victor Baldin Neder, Isabela Cristina Gomes Honório, Silvio Almeida-Junior

**Affiliations:** 1 Post Graduate Program in Animal Science, University of Franca, Franca, Brazil; 2 Laboratory of Biosciences and Health, Department of Biosciences, State University of Minas Gerais, Passos, Brazil; 3 Institute for Energy and Nuclear Research, São Paulo, Brazil; 4 Department of Agrarian and Earth Sciences, State University of Minas Gerais, Passos, Brazil

**Keywords:** Livestock, Multidrug resistance, Natural product, Veterinary phytotherapy

## Abstract

Bovine mastitis is one of the major challenges in dairy production, causing significant economic losses and emphasizing the need for alternatives to the indiscriminate use of antibiotics. Palo Santo (*Bursera graveolens*) essential oil (BEO) presents recognized biological properties that can be enhanced through nanoengineered delivery systems. This study aimed to chemically characterize the BEO of *B. graveolens*, develop a nanoemulsion containing the essential oil (BEO-NE), and evaluate its toxicological, antinociceptive, and antimicrobial activities against bovine mastitis–associated pathogens. The chemical composition of the BEO was determined by gas chromatography–mass spectrometry (GC–MS), identifying D-limonene (38.70%) as the major constituent. The BEO-NE was formulated using avocado oil as the oil phase and sodium alginate as a stabilizing agent. Toxicological and antinociceptive effects were assessed through in vivo assays, including acute toxicity, acetic acid–induced writhing, and the formalin test. Antimicrobial activity was evaluated by minimum inhibitory and bactericidal concentration assays against *Staphylococcus* spp., *Corynebacterium bovis*, *Streptococcus uberis*, and *Prototheca bovis*. The BEO-NE showed enhanced antimicrobial activity against *Staphylococcus aureus*, including MRSA isolates, while exhibiting lower efficacy against *C. bovis* and *S. uberis* compared with the free BEO, indicating pathogen-dependent selectivity. The formulation demonstrated a favorable safety profile, with no evidence of hepatotoxicity or behavioral alterations. The BEO also exhibited significant antinociceptive activity, mainly in the neurogenic phase of the formalin test (*p* < 0.05). In conclusion, the BEO-NE represents a promising strategy for controlling *S. aureus*, the main etiological agent of bovine mastitis, combining selective antimicrobial and antinociceptive effects.

## Introduction

Bovine mastitis is considered one of the most important diseases affecting the dairy industry, being responsible for substantial productive and economic losses. A significant reduction in milk yield often leads to the premature culling of cows, resulting in economic losses estimated at approximately 10% of the total herd revenue (Corrêa et al. 2024). These losses are even more pronounced in subclinical cases, in which early alterations are not readily detectable, thereby increasing expenditures related to veterinary services, antimicrobial therapies, and anti-inflammatory drugs (Paramasivam et al. 2023). This scenario has worsened over the years (Zhang et al. 2022), underscoring the urgent need for the development of more effective and safer strategies for the prevention and treatment of bovine mastitis.

Control of mastitis has traditionally relied predominantly on the use of antimicrobials (Tommasoni et al. 2023), an approach that has become progressively less effective due to the advancement of antimicrobial resistance. In this context (Eleodoro and Fagnani, 2023), reported that *Staphylococcus* spp. is the most frequently isolated pathogen in cases of subclinical bovine mastitis in Brazil, with an average prevalence of 49% among the analyzed samples, in addition to high levels of resistance to commonly used antimicrobials, particularly penicillin.

Coagulase-negative *Staphylococcus* spp. isolates exhibit high levels of multidrug resistance, with approximately 32% of strains showing simultaneous resistance to eight or more classes of antibiotics (Brito and Costa 2024). High resistance rates have also been reported for widely used antimicrobials, such as penicillin and ampicillin, as well as the emergence of methicillin-resistant *Staphylococcus aureus* (MRSA) strains in dairy herds (Khanal et al. 2022; de Oliveira et al. 2022).

This scenario is further aggravated by the biofilm-forming ability of mastitis-associated microorganisms, particularly *Staphylococcus* spp., a mechanism that contributes to infection persistence and reduces the effectiveness of conventional treatments (Vieira et al. 2024). It is estimated that antimicrobial therapies approved for intramammary use achieve clinical success rates ranging from 20 to 50%, reinforcing the need for complementary and preventive strategies (Li et al. 2023).

Milk is a widely consumed food worldwide and constitutes a dietary component for more than 80% of the global population, which corresponds to approximately six billion people (Algharib et al. 2024). In this context, the sanitary quality of milk plays a central role from a public health and food safety perspective. However, the intensive use of antimicrobials in dairy farming, particularly for the control of bovine mastitis, has been associated with the presence of drug residues in milk and the selection of resistant microorganisms, resulting in sanitary, economic, and environmental impacts (Zhang et al. 2022).

Within this framework**,** the prevention of bovine mastitis plays a fundamental role in dairy production systems. The adoption of appropriate management practices, combined with reduced exposure of the mammary gland to infectious agents, represents one of the main strategies for disease control (Tommasoni et al. 2023). Mastitis prevention is closely associated with factors such as milking time, equipment sanitation, herd health management, udder care, hand hygiene of milkers, and teat disinfection before and after milking (Sharun et al. 2021).

Given the limitations associated with the intensive use of antimicrobials and the importance of preventive measures, natural compounds have gained increasing attention as alternative strategies for the control of bovine mastitis. Among these compounds, essential oils stand out due to their antimicrobial, anti-inflammatory, and analgesic properties, and have been widely investigated against mastitis-associated microorganisms (Caneschi et al. 2023). However, their direct application presents limitations related to volatility, instability, and low solubility in aqueous media, factors that may compromise both their efficacy and safety (Nie et al. 2023).

To overcome these limitations, controlled delivery systems such as nanoemulsions have been proposed as a promising strategy for optimizing the use of essential oils. These systems improve the stability, bioavailability, and safety of bioactive compounds, while enhancing their biological activity, thereby enabling more effective veterinary applications (Polat Yemiş et al. 2022).

Among essential oils with therapeutic potential, the essential oil of *Bursera graveolens*, popularly known as Palo Santo (“madeira sagrada”), stands out. This tree, native to Mexico and Peru, contains phytochemical constituents of recognized pharmacological interest and has been traditionally used in folk medicine, with antimicrobial and analgesic properties described in the literature (Laurintino et al. 2023; Espinoza et al. 2021). However, studies integrating its antimicrobial activity against mastitis-associated pathogens, toxicological safety, and technological application in delivery systems remain scarce.

Therefore, the present study aimed to evaluate the antimicrobial, antinociceptive, anti-inflammatory, and toxicological activities of *Bursera* essential oil (BEO), both in its free form and formulated as a nanoemulsion, aiming at its potential application as a therapeutic alternative for the control of clinical and subclinical bovine mastitis.

## Methodology

### *Bursera graveolens* essential oil (BEO): obtaining and characterization

The extraction and characterization procedures were carried out according to protocols previously adopted and validated in earlier studies by the research group (Dos Santos et al. 2025a). The BEO was commercially obtained by wood distillation from the Brazilian company Via Aroma (Porto Alegre, Brazil; batch L6370007 v 01/26).

Qualitative characterization was carried out using gas chromatography (GC–MS) at the Federal University of Santa Catarina, using an AGILENT 7820A gas chromatograph equipped with an Rxi-5MS capillary column (30 m × 0.25 mm × 0.25 μm; Restek). The chromatographic conditions were as follows: initial column temperature of 50 °C (held for 0 min), increased at a rate of 3 °C/min to 220 °C. The injector temperature was set at 220 °C and operated in split mode (1:50). The detector (FID) temperature was maintained at 240 °C. The injection volume was 1 μL, with the essential oil diluted to 1.0% (v/v) in ethyl acetate.

### Preparation and characterization of the sodium alginate nanoemulsion containing *Bursera graveolens* essential oil

**Fig. 1:**
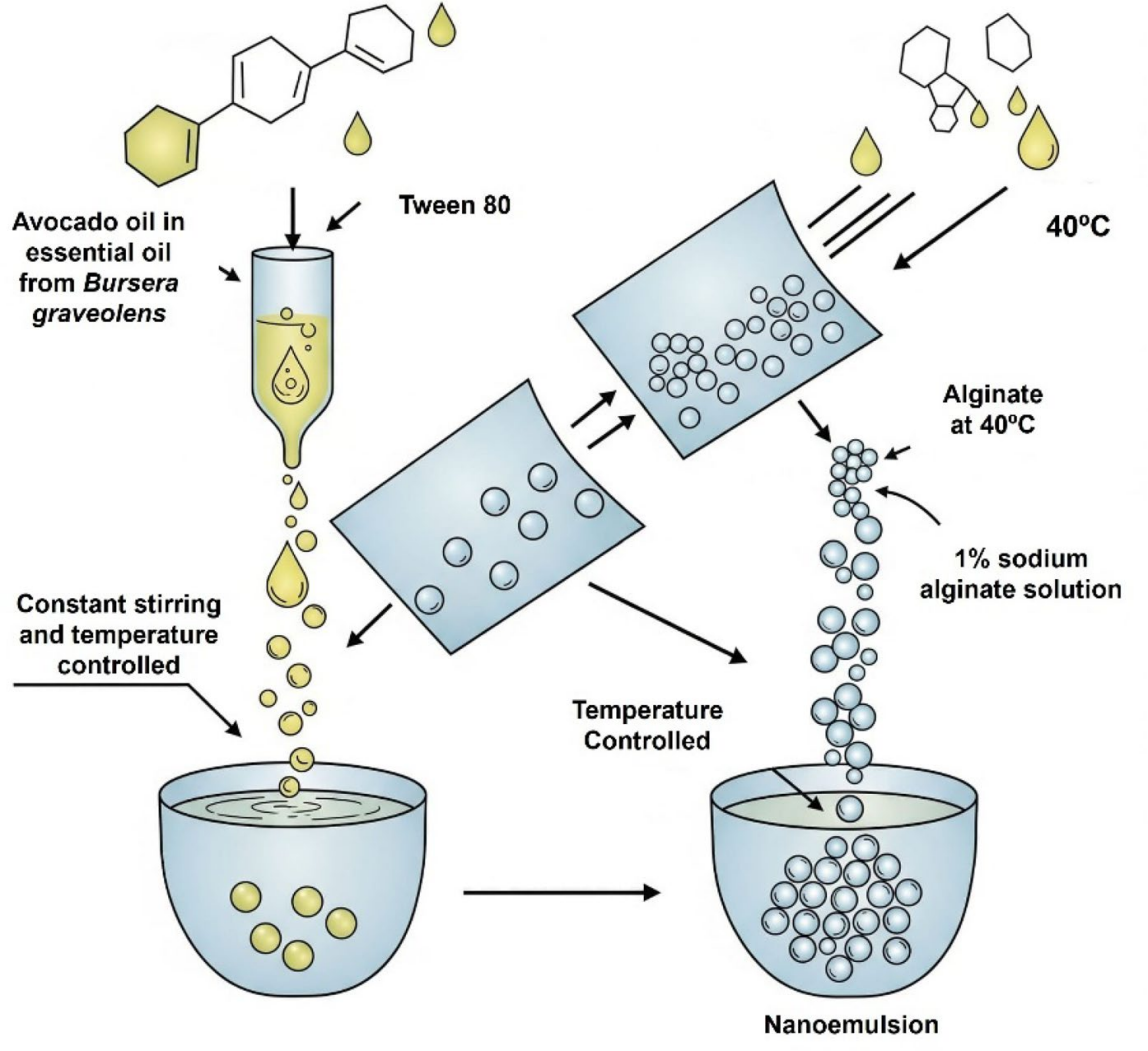
Development of *Bursera graveolens* essential oil nanoemulsion with sodium alginate by constant agitation

The nanoemulsion containing *B. graveolens* essential oil (BEO-NE) was developed using an adapted method (Fig. 1) based on emulsification under constant stirring and controlled temperature (Nie et al. 2023). Initially, the BEO was pre-solubilized in avocado oil, which served as the carrier oil phase. This oil phase was then incorporated into an aqueous solution containing Tween 80 (5%) as a non-ionic surfactant, under gentle magnetic stirring for 25 min, until a homogeneous pre-emulsion was obtained.

In parallel, a 1% (w/v) sodium alginate solution was prepared by dissolving the polymer in ultrapure water under constant stirring (400 rpm), maintained at 40 °C until complete solubilization (Polat Yemiş et al. 2022). The BEO containing pre-emulsion was then slowly added to the alginate solution under continuous stirring, resulting in a system with a final oil phase content of 30% (v/v).

After complete incorporation of all components, the formulation was subjected to ultrasonication using an ultrasonic homogenizer to promote droplet size reduction and nanoemulsion formation. System homogeneity was qualitatively assessed by optical microscopy.

The mean hydrodynamic diameter (*Z*_avg_), polydispersity index (PDI), transmittance (%), and diffusion coefficient (*D*) were determined by Dynamic Light Scattering (DLS) (Baranauskaite et al. 2021) using a Litesizer 500 instrument (Anton Paar GmbH, Austria), equipped with a 658 nm laser and detection angles of 90° and 175°, under controlled temperature conditions (25 ± 0.1 °C). BEO-NE samples were diluted at a ratio of 1:80 (v/v) in ultrapure water filtered through a 0.22 μm membrane prior to analysis. Nanoemulsion stability was evaluated at the initial time point (0 days) and after 90 days of storage under refrigeration (5 ± 2 °C), with results expressed as mean ± standard deviation. Statistical analysis was performed using the Student’s t-test to compare the means between the evaluated time points, considering a significance level of *p* < 0.05.

### In vitro antimicrobial activity

#### Microorganisms

The experimental protocol was approved by the Animal Ethics Committee of the State University of Minas Gerais (approval no. 03/2025, March 26, 2025). Milk samples were collected on demand from dairy farms located in southern Minas Gerais, Brazil. Animals were selected based on the presence of clinical or subclinical mastitis, with no other apparent health disorders. Clinical mastitis was diagnosed by farm veterinarians based on clinical examination of the mammary gland and milk appearance, whereas subclinical mastitis was confirmed using the California Mastitis Test, based on somatic cell count in milk samples.

Milk samples were collected using sterile gloves and sterile containers. The first milk streams were discarded, and approximately 10 mL of milk was collected into properly identified sterile tubes and transported on ice at 4 °C to the Laboratory of Biosciences and Health at the University of Minas Gerais State, Passos campus. Bacteriological isolation and identification of mastitis-associated pathogens were performed using standard bacteriological diagnostic methods (Tomanić et al. 2022). Identification of the isolates was performed based on colony morphology, Gram staining, and standard biochemical tests, including catalase and coagulase assays for *Staphylococcus* spp., as well as additional tests according to each bacterial group, following established microbiological protocols.

Ten identified strains were selected for preclinical assays, including Gram-negative bacteria (*Escherichia coli* and *Serratia marcescens*), Gram-positive bacteria (*Corynebacterium bovis*, *Staphylococcus epidermidis*, *Staphylococcus sciuri*, *Streptococcus agalactiae*, *Streptococcus uberis*, and two *Staphylococcus aureus* strains [1 and 2]), as well as the achlorophyllous alga *Prototheca bovis*.

The strains were cultured on *Mueller–Hinton* agar (LaborClin, batch no. 230224036) and incubated at 37 °C for 24 h. A single colony from each plate was suspended in 10 mL of phosphate-buffered saline until turbidity equivalent to a 0.5 McFarland standard (1.5 × 10^8^ CFU/mL), according to Clinical and Laboratory Standards Institute (CLSI) guidelines (Perez et al. 2020).

#### Antimicrobial susceptibility test by disk diffusion

Antimicrobial activity was evaluated using the disk diffusion assay (Orszulik 2022). Sterile filter paper disks (6.35 mm diameter; 80 g) were impregnated with the treatments: BEO (15 µg) and BEO-NE (15 µg). As positive controls, antimicrobial agents from different classes were used at standardized concentrations according to Clinical and Laboratory Standards Institute (CLSI) guidelines, including the β-lactams AMC (amoxicillin/clavulanic acid, 30 µg) and AMP (ampicillin, 30 µg), the aminoglycoside GEN (gentamicin, 30 µg), the phenicol CL (chloramphenicol, 30 µg), the fluoroquinolone CIP (ciprofloxacin, 30 µg), and the tetracycline TET (tetracycline, 30 µg).

Plates were incubated under aerobic conditions at 35 ± 1 °C for 18 ± 2 h. After incubation, inhibition zone diameters were measured from the underside of the plates against a dark background under reflected light. Based on the results, microorganisms were classified as susceptible (S), intermediate (I), or resistant (R).

Interpretation of antimicrobial susceptibility for antibiotics followed CLSI guidelines (Humphries et al. 2021). For BEO and BEO-NE treatments, results were evaluated according to criteria described in previous studies involving natural products and essential oils (Arbab et al. 2022).

#### Broth microdilution susceptibility test

The antimicrobial susceptibility profile for determination of the minimum inhibitory concentration (MIC) and minimum bactericidal concentration (MBC) was assessed using the broth microdilution method, performed in sterile 96-well microtiter plates according to previously established protocols of the research group (Damasceno et al. 2026).

Inocula were standardized to a final concentration of 5 × 10^5^ CFU/mL per well. BEO and BEO-NE were previously solubilized in dimethyl sulfoxide (DMSO 0,1%) and brain heart infusion broth and tested at concentrations ranging from 0.195 to 200 µg/mL. Plates were incubated at 37 °C for 24 h under aerobic conditions, maintaining a final DMSO concentration ≤ 5% (v/v), which was used as the negative control. Microbial viability was assessed by the addition of 30 µL of 0.02% resazurin solution (Sigma-Aldrich) to each well (Sarker et al. 2007).

Minimum bactericidal concentration (MBC) was determined by subculturing 10 µL aliquots from wells showing no visible growth onto blood agar base supplemented with 5% defibrinated sheep blood. MBC was defined as the lowest concentration at which no bacterial growth was observed. MBC values higher than MIC were indicative of a bacteriostatic effect, whereas coincident MIC and MBC values were characteristic of a bactericidal effect (Leandro et al. 2016).

### In vivo assays

#### Animals

All procedures involving animals were reviewed and approved by the Institutional Animal Care and Use Committee (CEUA-IPEN, protocol no. 74/24), in accordance with national and international guidelines for animal experimentation. A total of 78 male BALB/c mice were obtained from the Animal Facility of the Nuclear and Energy Research Institute (IPEN), São Paulo, Brazil. The experiments were conducted in an experimental room under controlled temperature (23 ± 2 °C) and humidity (50 ± 10%), with a 12 h light/dark cycle and free access to food and water (ad libitum).

#### Toxicological evaluation of the essential oil and the essential oil-loaded nanoemulsion

The toxicological safety of the BEO and BEO-NE was assessed using the open-field test, with analysis of locomotor activity and observation of general clinical signs. Male BALB/c mice were randomly distributed into experimental groups (n = 6), including a negative control group and groups treated with the highest previously defined doses of BEO (50 mg/kg) and BEO-NE (15 mg/kg).

The open-field test (Almeida Junior 2019) was performed using a plastic arena measuring 45 × 45 × 20 cm, with the floor divided into nine equal squares (15 × 15 cm). Spontaneous locomotor activity was evaluated by counting the number of squares crossed with all four paws during 6 min sessions, before and after treatment administration, and was considered a behavioral index.

After the open-field test, animals were monitored for signs of acute toxicity during the first 4 h, including changes in locomotion, behavior (agitation, lethargy, and aggressiveness), respiratory pattern, salivation, lacrimation, peripheral cyanosis, piloerection, and mortality. The same animals were used to assess acute toxicological potential according to OECD guideline 425 (OECD, 425 2022). Body weight variation (Almeida Junior et al. 2021) was monitored throughout the experimental period as an indicator of systemic toxicity. In addition, animals were observed daily for 14 days to record delayed clinical signs and mortality.

On experimental day 15, prior to euthanasia, animals were sedated with low doses of sodium thiopental administered intraperitoneally until loss of consciousness, in order to minimize stress and pain. Blood samples were then collected by intracardiac puncture for determination of serum alanine aminotransferase (ALT) and aspartate aminotransferase (AST) activities, used as biochemical markers of potential hepatic injury (Junior et al. 2020).

Subsequently, animals were euthanized by intraperitoneal administration of sodium thiopental (0.84 g). A post-mortem examination was performed to investigate macroscopic alterations of the gastrointestinal tract, aiming to evaluate possible irritant or caustic effects associated with the tested formulations.

#### Evaluation of antinociceptive and anti-inflammatory activity

The antinociceptive and anti-inflammatory potential of BEO and BEO-NE was evaluated using the formalin test and the acetic acid–induced abdominal writhing test, as described in the literature (Almeida Junior 2019).

Male BALB/c mice were randomly assigned to experimental groups (n = 6), including: a negative control group; a group treated with a standard analgesic drug (morphine, 2.5 mg/kg, intraperitoneally), administered 30 min before the tests; a group treated with a standard anti-inflammatory drug (indomethacin, 10 mg/kg, orally by gavage), administered 60 min before the tests; and groups treated with BEO (25 and 50 mg/kg, orally by gavage) and BEO-NE (15 mg/kg, orally by gavage), administered 60 min prior to the assays.

In the formalin test, 20 µL of 2.5% formalin was injected into the dorsal surface of the right hind paw, 1 h after treatment administration. Animals were immediately placed in individual observation chambers, and nociceptive behavior was recorded as the total time (in seconds) spent licking, biting, or shaking the injected paw during the first 5 min (Phase I—neurogenic pain) and between 15 and 30 min after injection (Phase II—inflammatory pain), using a digital stopwatch (Almeida Junior et al. 2021).

The abdominal writhing test was performed by intraperitoneal administration of 0.6% acetic acid (0.1 mL/10 g body weight), 60 min after treatment administration. Following injection, animals were observed for 20 min, and the total number of abdominal writhes, characterized by abdominal contractions followed by extension of the hind limbs, was recorded (Almeida Junior 2019).

### Statistical analysis

Statistical analysis was performed using GraphPad Prism software, version 8.0.1. Comparisons between data obtained before and after treatments were conducted using Student’s t-test. For the remaining analyses, comparisons among experimental groups were performed using one-way analysis of variance (ANOVA), followed by Tukey’s post hoc test for multiple comparisons. The presence of outliers was assessed using the ROUT test, and outliers were excluded when identified. Differences were considered statistically significant when *p* < 0.05.

## Results

### Characterization of the chemical constituents of *Bursera graveolens* essential oil

**Fig. 2:**
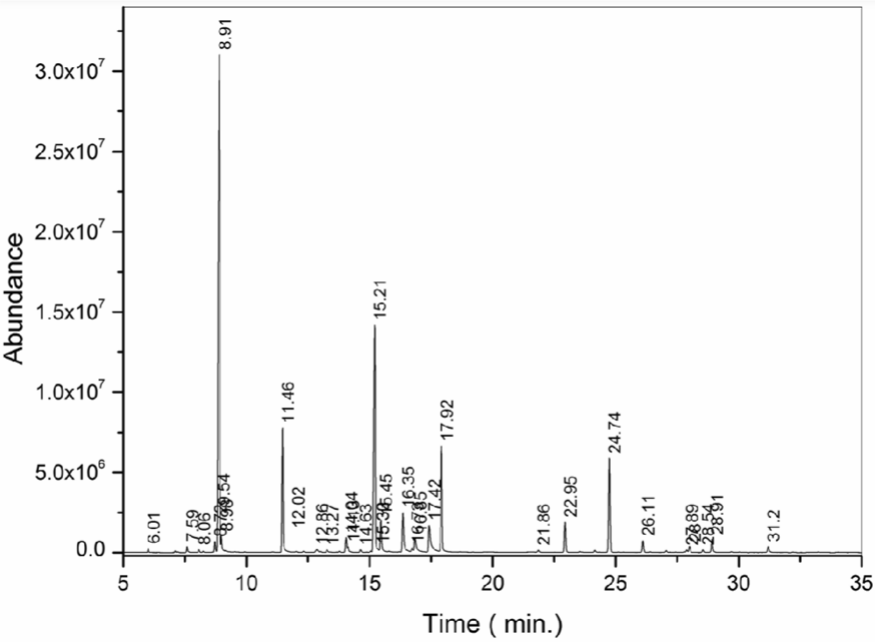
Qualitative characterization of the volatile constituents of *Bursera graveolens* essential oil performed by gas chromatography coupled to mass spectrometry

Chromatographic analysis (Fig. 2) enabled the identification of the main constituents of the BEO (Table 1), with D-limonene identified as the major compound (RT 8.91 min; 38.70%), followed by α-terpineol (RT 15.21 min; 17.31%). Other compounds were detected at lower relative abundances, including oxygenated monoterpenes and sesquiterpenes, reflecting a chemically diverse profile. Minor constituents accounted for 0.84% of the sample, whereas 1.42% of the detected compounds could not be identified.

**Table 1:** Identification of volatile compounds of *Bursera graveolens* essential oil by gas chromatography coupled to mass spectrometry

TR (min)	Compound	Percentage (%)
6,01	α-Pinene	0,19
7,59	Myrcene	0,32
8,72	p-Cymene	0,65
8,91	D-Limonene	38,70
8,98	Eucalyptol	1,11
9,54	β-Ocimene	0,14
11,47	Linalool	7,44
12,02	Fenchol	0,13
12,87	cis-p-Mentha-2,8-dien-1—ol	0,33
13,27	β-Terpineol	0,21
14,04	Menthofuran	1,00
14,13	Borneol	0,49
14,63	Terpinen-4—ol	0,20
15,21	α-Terpineol	17,31
15,46	γ-Terpineol	2,43
16,35	trans-Carveol	3,34
16,73	cis-p-mentha-1(7),8-dien-2—ol	0,36
16,85	cis-Carveol	1,47
17,42	Carvone	3,28
17,92	Linalyl acetate	6,56
21,86	α-Cubebene	0,30
22,95	α-Copaene	1,99
24,15	Longifolene	0,17
24,75	Caryophyllene	6,34
26,12	α-Humulene	0,81
27,06	γ-Muurolene	0,15
28,00	α-Muurolene	0,46
28,54	γ-Muurolene	0,21
28,91	δ-Cadinene	1,08
31,20	Caryophyllene oxide	0,56
–	Minority compounds	0,84
–	Unidentified compounds	1,42

### Preparation and physicochemical characterization of the nanoemulsion

**Fig. 3:**
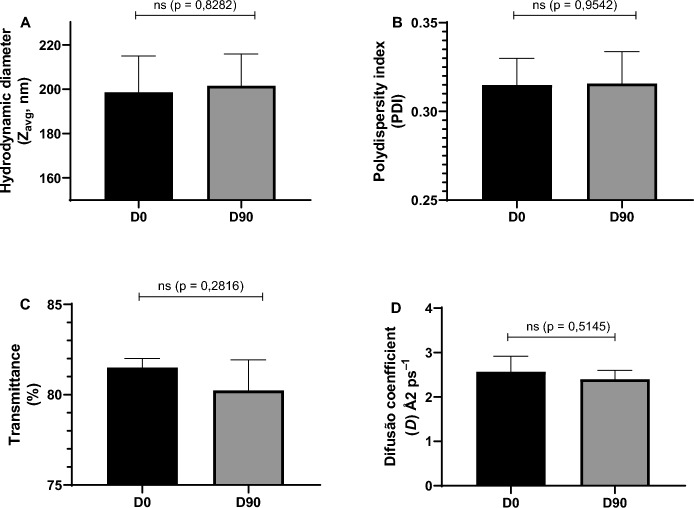
Physicochemical characterization of sodium alginate nanoemulsion containing *Bursera graveolens* essential oil before (D0) and after 90 days (D90) of storage under refrigeration (5 ± 2 °C), including mean hydrodynamic diameter (Z_avg_) (**A**), polydispersity index (PDI) (**B**), transmittance (%) (**C**) and diffusion coefficient (*D*) (**D**). Data are presented as mean ± standard deviation

The BEO-NE exhibited a Z_avg_ of 198.6 ± 16.41 nm at the initial time point (Fig. 3A), remaining essentially unchanged after 90 days of storage at 5 ± 2 °C (201.6 ± 14.33 nm), indicating good physicochemical stability over the evaluated period. The polydispersity index (PDI) (Fig. 3B) also remained stable, with values of 0.31 ± 0.02 before storage and 0.32 ± 0.02 after 90 days, suggesting maintenance of a homogeneous droplet size distribution.

Transmittance values (Fig. 3C) showed a slight decrease over time, ranging from 81.50 ± 0.50% to 80.23 ± 1.69%, without evidence of physical instability or phase separation. Consistently, the diffusion coefficient (D) (Fig. 3D) exhibited a discrete reduction after the storage period (from 2.567 ± 0.351 Å^2^ ps^−1^ to 2.400 ± 0.200 Å^2^ ps^−1^), which is compatible with the small increase observed in the hydrodynamic diameter of the droplets.

### In vitro antimicrobial activity

The antimicrobial activity (Table 2), assessed by the disk diffusion method and interpreted according to CLSI criteria, revealed a high level of resistance to conventional antimicrobials among the tested microorganisms. Predominant resistance was observed across major antibiotic classes, including β-lactams, fluoroquinolones, aminoglycosides, and tetracyclines, particularly among isolates of *C. bovis*, *P. bovis*, *S. marcescens*, and *S. aureus*.

**Table 2:** Profile of antimicrobial susceptibility and resistance of the microorganisms evaluated against conventional antibiotics, *Bursera graveolens* essential oil (BEO) and essential oil-containing nanoemulsion (BEO-NE), determined by the disk diffusion method

Microorganism	*AMC*	*AMP*	*CL*	*CIP*	*GEN*	*TET*	*BEO*	*BEO-NE*
*Corynebacterium bovis*	R	R	R	R	R	R	S	R
*Escherichia coli*	R	S	S	R	S	S	R	R
*Prototheca bovis*	R	R	R	R	R	R	S	S
*Serratia marcescens*	R	R	S	R	R	R	S	S
*Staphylococcus aureus (1)*	R	R	R	I	R	R	S	S
*Staphylococcus aureus* (2)	R	R	R	R	R	R	S	S
*Staphylococcus epidermidis*	S	S	S	S	S	R	S	S
*Staphylococcus sciuri*	S	S	S	S	S	S	R	S
*Streptococcus agalactiae*	R	R	R	S	S	S	R	S
*Streptococcus uberis*	R	R	S	S	S	S	S	R

Data on antimicrobial activity are presented, determining resistance (R), sensitivity (S) and indeterminate (I) profiles according to CLSI

*AMC* amoxicillin/clavulanic acid (β-lactam), *AMP* ampicillin (β-lactam), *CL* chloramphenicol (phenicol), *CIP* ciprofloxacin (fluoroquinolone), *GEN* gentamicin (aminoglycoside), *ETT* tetracycline (tetracycline)

The use of oxacillin (data not shown) for phenotypic characterization of *S. aureus* confirmed resistance in the *S. aureus* (2) isolate, indicating a methicillin-resistant *S. aureus* (MRSA) phenotype in this specific strain.

In contrast, the BEO exhibited antimicrobial activity against most of the evaluated microorganisms, including isolates displaying multidrug-resistant profiles. BEO-NE demonstrated antimicrobial activity comparable to or greater than that of BEO, showing susceptibility against microorganisms resistant to conventional antibiotic classes, including *S. aureus* isolates.

The determination of the MIC and MBC (Table 3) demonstrated relevant antimicrobial activity of the BEO, with greater efficacy against Gram-positive microorganisms. Notably, *S. epidermidis*, *S. uberis*, and *C. bovis* exhibited MIC and MBC values ≤ 3.125 mg/mL. In contrast, *S. sciuri*, *S. agalactiae*, and the Gram-negative microorganism *E. coli* showed low sensitivity to the essential oil, with MIC and MBC values exceeding 200 mg/mL.

**Table 3:** Minimum inhibitory concentration (MIC) and minimum bactericidal concentration (MBC) of the essential oil and nanoemulsion containing *Bursera graveolens* essential oil against microorganisms of clinical and veterinary interest, determined by the broth microdilution method

	MIC (mg/mL)	MBC (mg/mL)
*Bursera graveolens* essential oil		
*Corynebacterium bovis*	3,125	3,125
*Escherichia coli*	> 200	> 200
*Prototheca bovis*	1,56	1,56
*Serratia marcescens*	12,5	12,5
*Staphylococcus aureus*	12,5	12,5
*Staphylococcus aureus* (MRSA)	6,25	12,5
*Staphylococcus epidermidis*	3,125	6,25
*Staphylococcus sciuri*	> 200	> 200
*Streptococcus agalactiae*	> 200	> 200
*Streptococcus uberis*	3,125	3,125
*Bursera graveolens* nanoemulsion		
*Corynebacterium bovis*	> 200	> 200
*Escherichia coli*	> 200	> 200
*Prototheca bovis*	1,56	3,12
*Serratia marcescens*	1,56	3,125
*Staphylococcus aureus*	3,125	3,125
*Staphylococcus aureus* (MRSA)	3,125	6,25
*Staphylococcus epidermidis*	0,78	1,56
*Staphylococcus sciuri*	1,56	3,125
*Streptococcus agalactiae*	1,56	1,56
*Streptococcus uberis*	> 200	> 200

BEO-NE showed a marked improvement in antimicrobial activity compared to the free essential oil, with a consistent reduction in MIC and MBC values for most of the evaluated microorganisms. Enhanced activity was observed against *S. aureus*, including the MRSA isolate, as well as against *S. sciuri*, *S. epidermidis*, *S. agalactiae*, and *S. marcescens*, indicating the potential of the formulation to increase essential oil bioavailability and antimicrobial efficacy.

Antimicrobial activity against *P. bovis* remained high for both formulations. Conversely, *E. coli*, *S. uberis*, and *C. bovis* remained resistant to the nanoemulsion under the tested conditions.

Additionally, sodium alginate (1%), Tween 80 solution (5%), and avocado oil, used as excipient and vehicle controls for the formulations, were evaluated individually against the tested microorganisms. None of these substances exhibited detectable antimicrobial activity under the experimental conditions, as no inhibition zones or reductions in microbial growth were observed.

### In vivo assays

#### Toxicological evaluation

Following the open-field test, no evidence of acute toxicity was observed in animals treated with the BEO or the BEO-NE. During the initial 4 h observation period, no alterations were detected in locomotor activity, behavior (agitation, lethargy, or aggressiveness), respiratory pattern, salivation, lacrimation, or signs of peripheral cyanosis or piloerection. In addition, no mortality or indications of toxicity were recorded in any of the evaluated groups after the observation period established by OECD guideline 425.

**Fig. 4:**
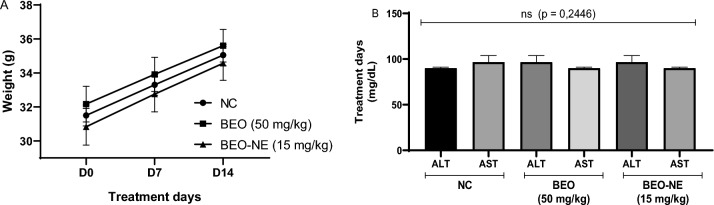
Evaluation of the toxicological safety of *Bursera graveolens* essential oil (BEO) and essential oil-containing nanoemulsion (BEO-NE) in Balb/c mice. Variation in body weight (**A**) of the animals over the experimental period, with no statistically significant differences between the groups (*p* = 0.2334). Serum levels of alanine aminotransferase (ALT) and aspartate aminotransferase (AST) (**B**), used as markers of liver toxicity, showed no significant changes after treatment (*p* = 0.2446). The data are presented as mean ± standard deviation, according to OECD protocol 425

No statistically significant differences in body weight variation (3.6 ± 0.3 mg) were observed among the experimental groups (*p* = 0.2334), as illustrated in Fig. 4A. Similarly, no significant alterations were detected in serum ALT and AST levels (Fig. 4B), indicating the absence of hepatic toxicity, with mean concentrations remaining stable across groups (*p* = 0.2446).

**Fig. 5:**
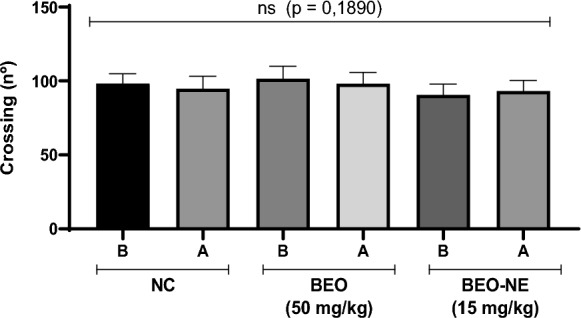
Safety and toxicity data of *Bursera graveolens* essential oil (BEO) and essential oil-containing nanoemulsion (BEO-NE) in Balb/c mice in the open field assay. The negative control group (NC), the group treated with BEO at a dose of 50 mg/kg, and BEO-NE at a dose of 15 mg/kg are presented. Behavioral parameters were evaluated before (**B**) and after (**A**) treatment, and statistical analysis considering a significance level of *p* < 0.05

Consistent with the previous observations, the open-field test assessing the number of crossings (Fig. 5) revealed no significant differences among the experimental groups (*p* = 0.1890), with mean values close to 100 across all evaluated conditions. *Post-mortem* macroscopic examination of the gastrointestinal tract revealed no morphological alterations in animals treated with the BEO or the BEO-NE. No signs compatible with irritant or caustic effects were observed, including hyperemia, edema, ulcerations, areas of necrosis, hemorrhage, or apparent structural changes in the gastrointestinal mucosa. These findings further support the safety profile of the evaluated formulations, indicating the absence of local gastrointestinal toxicity under the experimental conditions employed.

#### Antinociceptive and anti-inflammatory activity

**Fig. 6:**
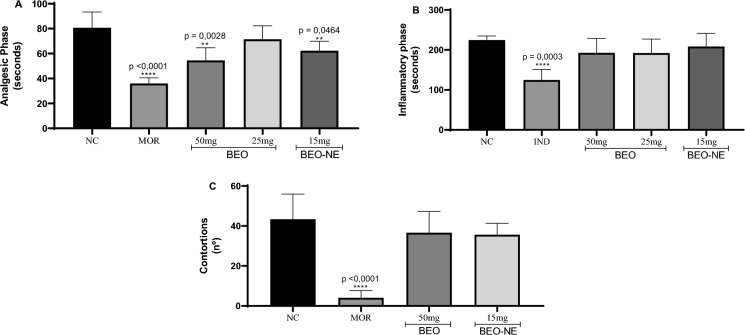
Antinociceptive and anti-inflammatory potential assays of *Bursera graveolens* essential oil (BEO) and essential oil-containing nanoemulsion (BEO-NE) on the Formalin assay in mice presenting the neurogenic pain phase (**A**) and inflammatory pain (**B**), in addition to the analgesic potential by the abdominal contortion assay (**C**). * statistical difference when compared to the negative control

In the formalin-induced nociception assay, both the BEO and the BEO-NE reduced nociceptive behavior during both phases of the test. In Phase I, which reflects neurogenic pain (Fig. 6A), BEO administered at 50 mg/kg produced a marked reduction in licking time compared with the control group. Notably, BEO-NE (15 mg/kg) exerted a consistent antinociceptive effect even at a lower dose, indicating enhanced efficacy of the nanoemulsified formulation. These reductions were statistically significant (BEO, *p* = 0.0028; BEO-NE, *p* = 0.0464). In Phase II, associated with inflammatory pain (Fig. 6B), both BEO and BEO-NE reduced the nociceptive response time, suggesting an effect related to modulation of the inflammatory process; however, this reduction did not reach statistical significance (*p* > 0.05).

Similarly, in the acetic acid–induced abdominal writhing test (Fig. 6C), a decrease in the mean number of writhes was observed in animals treated with BEO and BEO-NE compared with the negative control, although this reduction was not statistically significant (*p* > 0.05).

## Discussion

The technological efficiency of the nanoemulsion partially explains the superior performance observed in some analgesic (Teixé-Roig et al. 2022) and antimicrobial assays (Polat Yemiş et al. 2022). Nanoengineered delivery systems are known to enhance the solubility and bioavailability of terpenes, while protecting bioactive compounds from volatilization and oxidative degradation, thereby increasing their persistence within the experimental matrix (Kaspute et al. 2025).

Nanoemulsion-based delivery systems, such as BEO-NE, are well-established strategies in pharmaceutical and veterinary nanotechnology (Polat Yemiş et al. 2022). The colloidal stability observed in the present formulation, evidenced by the absence of aggregation phenomena, is consistent with the expected behavior of systems containing surfactants and hydrophobic natural compounds (Kogan and Garti 2006; Nguyen et al. 2024).

Preclinical safety was supported by the acute toxicity evaluation of both the BEO and BEO-NE, as well as by the absence of alterations in hepatic biomarkers (ALT and AST) and body weight gain. The limited availability of toxicological data on *Bursera graveolens* essential oil in the literature (Laurintino et al. 2023) highlights the novelty of the present study. In contrast, Wojtunik-Kulesza et al. (2022) reported a favorable safety profile for moderate doses of monoterpenes such as D-limonene and α-terpineol, which were identified as major constituents of the BEO. The lack of behavioral or hepatic alterations observed herein further supports the physiological compatibility of *B. graveolens* essential oil, particularly when associated with microencapsulation strategies, which may modulate absorption kinetics and reduce local irritation.

Antinociceptive potential was primarily evidenced during Phase I (neurogenic pain) of the formalin assay, with effects comparable to those observed for opioid analgesics. This response suggests a modulatory action on transient receptor potential channels, leading to attenuation of nociceptive signaling and a reduction in peripheral inflammatory mediators, as previously demonstrated by Kaimoto et al. (Kaimoto et al. 2016) using D-limonene, the major constituent identified in the present study. In addition, α-terpineol has been reported to exert analgesic effects and to reduce the production of pro-inflammatory cytokines, which may synergistically contribute to the observed antinociceptive activity (Soleimani et al. 2019).

Previous studies involving BEO have also reported cytokine modulation, which may underlie its efficacy in the formalin-induced nociception model. Although Phase I of the formalin test is classically associated with direct activation of nociceptors, inflammatory mediators such as TNF-α, IL-17A, IL-23, and IL-8 may participate in early nociceptive signaling and peripheral sensitization, thereby influencing the neurogenic component of pain (Wang et al. 2022).

In the inflammatory phase of the formalin assay and in the acetic acid–induced writhing test, neither the BEO nor the BEO-NE exhibited significant antinociceptive activity. This finding supports the hypothesis of a preferential interaction of the BEO with central analgesic mechanisms associated with Phase I (neurogenic pain), rather than with peripheral inflammatory pathways involved in Phase II. This hypothesis is supported by previous investigations involving monoterpene-rich essential oils, which report predominant central modulation of nociceptive signaling (Kaimoto et al. 2016; Guimarães et al. 2013).

The antimicrobial findings reveal a complex activity profile of both BEO and BEO-NE against pathogens associated with bovine mastitis. Previous studies have demonstrated that encapsulation strategies can enhance the antimicrobial efficacy of essential oils (Polat Yemiş et al. 2022). In the present study, despite the BEO-NE containing only 30% of *B. graveolens* BEO, improved antimicrobial activity was observed against *S. marcescens*, *S. sciuri*, and *S. agalactiae*. This enhancement is likely attributable to the nanoemulsion system, which facilitates closer interaction between the bioactive compounds and the bacterial cell wall, thereby potentiating antimicrobial effects (Singh et al. 2017).

Both BEO and BEO-NE exhibited significant antimicrobial activity against *S. aureus*, including MRSA. These results underscore the translational potential of these formulations for controlling major etiological agents of bovine mastitis, particularly multidrug-resistant microorganisms (Khanal et al. 2022). The observed efficacy further reinforces the relevance of natural product–based approaches as strategic alternatives in veterinary health and livestock disease management (Li et al. 2023).

The reduced antimicrobial activity of BEO-NE against *C. bovis* and *S. uberis*, when compared with the free BEO, may be attributed to the partitioning behavior of hydrophobic constituents within the oil phase of the nanoemulsion system (Asres et al. 2025). D-limonene and other terpene compounds, when solubilized within nanometric droplets or sequestered by Tween 80 micelles, may exhibit a sustained-release profile, resulting in a lower concentration of bioactive compounds available during the incubation period and, consequently, below the MIC required for antimicrobial activity. Similar behavior has been reported for encapsulated BEO, in which antimicrobial efficacy was highly dependent on the structural characteristics of the target bacterial species (Sousa et al. 2022).

Gram-negative bacteria generally display lower susceptibility to BEO due to the presence of an outer membrane rich in lipopolysaccharides, which acts as a selective permeability barrier against hydrophobic compounds (Dos Santos et al. 2025a). This structural feature particularly restricts the activity of hydrocarbon monoterpenes and is consistent with the absence of antimicrobial activity observed against *E. coli* in the present study.

Although there is evidence that D-limonene, a terpene hydrocarbon, can exert antimicrobial effects through disruption of the bacterial cell membrane and leakage of intracellular components, its isolated activity is typically weak and highly dependent on elevated concentrations, oxidative transformation, or specific formulation strategies (Gupta et al. 2021). In contrast, studies reporting antimicrobial activity of essential oils against *E. coli*, such as those involving *Lippia graveolens*, *Thymus vulgaris*, and *Origanum vulgare*, have primarily attributed this efficacy to high levels of phenolic monoterpenes, including thymol and carvacrol (Lara et al. 2016).

BEO-NE exhibited antimicrobial activity against *Serratia marcescens*, with MIC and MBC values lower than those observed for *E. coli*. This finding suggests that interspecific differences in outer membrane permeability, combined with the nanoengineered formulation (da Silva et al. 2023; Brito et al. 2024), may enhance the interaction between bioactive compounds and the bacterial cell, thereby broadening the antimicrobial spectrum of the BEO. Recent studies have emphasized the marked variability in the efficacy of plant extracts and BEO against mastitis-associated pathogens and highlighted the critical role of formulation strategies in determining antimicrobial performance (Souza et al. 2024).

The antibiotic susceptibility profile of the *P. bovis* isolate evaluated in this study revealed extensive resistance. Previous investigations by Morandi et al., (2016) have reported high resistance of this genus to conventional antimicrobials, particularly β-lactams, fluoroquinolones, and sulfonamide/trimethoprim combinations, which is consistent with the findings reported here. Rodríguez et al., (2025) described susceptibility of specific strains to aminoglycosides, such as gentamicin. Phenolic and monoterpenoid compounds have been shown to compromise cell wall and membrane integrity, promote uncontrolled ion influx, and disrupt cellular energy metabolism, mechanisms that may contribute to the activity observed against *P. bovis* (Grzesiak et al. 2018).

Monoterpenes were identified as the predominant constituents of the BEO (Laurintino et al. 2023). These results are consistent with studies conducted by Sosa et al., (2023), which identified D-limonene as the primary phytochemical marker responsible for the characteristic aroma of the oil, followed by α-terpineol. Together, these findings reinforce the biological relevance of monoterpenes, particularly with respect to their antimicrobial and analgesic properties (Bakkali et al. 2008).

Collectively, these results underscore the biotechnological value of encapsulated essential oils and align with current trends aimed at developing alternative natural antimicrobial agents to mitigate bacterial resistance and improve animal health (Movahedi et al. 2024). Further investigations involving in vivo models of bovine mastitis (Vieira et al. 2024), targeted mechanistic studies, and evaluations under conditions that closely resemble production systems are warranted to expand understanding of the observed effects. Such approaches will facilitate formulation refinement, dose optimization, and consolidation of practical applicability in bovine mastitis control, particularly against multidrug-resistant pathogens (Caneschi et al. 2023; Nie et al. 2023). Importantly, the present study does not propose replacing antibiotics, but rather supports the complementary use of essential oil–based formulations within integrated therapeutic strategies.

In conclusion, the essential oil of *Bursera graveolens* exhibits a robust chemical profile, characterized by the predominance of D-limonene, and demonstrates feasibility for incorporation into nanoemulsion-based delivery systems. Although the system showed variability in efficacy among the tested bacterial strains, the marked enhancement of activity against *S. aureus* supports the application of nanotechnology as a strategy to optimize the treatment of bovine mastitis. The identified neurogenic antinociceptive effect further complements the pharmacological profile of the formulation. Future studies addressing long-term stability and in vivo safety assessments in bovine mammary glands are required to consolidate this nanoemulsion as a sustainable alternative to conventional antibiotic therapy.

## Data Availability

No datasets were generated or analysed during the current study.
